# Plea for Prisoners

**Published:** 1889-04

**Authors:** S. H. Preston


### PLEA FOR PRISONERS.

BY S. H. PRESTON.

Readers, did you ever commit an offence against the person or property of a fellow being? Reflect long and well before you reply. Rummage your memory, recall the daily deeds of your life, ransack the archives of your conscience.

Dare you declare you never did? If so, you are either a colossal liar, or a saint out of your sphere. If you never committed an act that could be construed into a violation of the criminal code, you are a paragon of human perfection, and there was a mighty mistake in placing you upon this planet.

Very likely you never inflicted an injury for which you were legally liable, never maliciously committed a crime. Well, that is something to settle with your conscience, but were you accused in court, it would devolve upon twelve other gentlemen to decide. The injury proved, the malicious motive would be presumed. The law explicitly implies a guilty intent for every injurious act. The accused must clearly and conclusively show the absence of such intent to escape the prescribed penalty. He can only do this by circumstantial evidence, as a person's purposes do not admit of positive proof.

Now, it may be said that sufficient evidence could be adduced to show that every individual, in some sort of way, had injured the person or property of another. Then, the criminal intent being inferred, all could be considered criminals. Those fortunate enough not to be found out and those able to exculpate themselves from premeditated malice, of course, escape conviction.

Admit that there may be such a wonder in this wicked world as an immaculate man. Even he would be liable to be caught in the cobwebs of the criminal code. The same sort of circumstantial evidence which sometimes saves a guilty wretch from the gallows may doom an innocent man. It is just as the gentlemen in the jury box consider the circumstances in the case. A man may become branded as a criminal by merely making a blunder—by picking up the wrong parcel in a public place, or by entering the wrong house on a block and being unable to show that he did not do so burglariously. He may be convicted of murder for committing a careless act, as accidently causing the death of another ; for a justifiable act, as taking life in defence of his own ; or even for a commendable act, as striving to save a suicide and being charged with killing the person he would save ; the exculpating circumstances in each case not appearing in proof. So that no man, however circumspect his conduct, has an absolute assurance that he will not be condemned as a criminal, while every real rogue, through favoring circumstances, may slip scot free, right through the loopholes of the law.

While many innocent are unjustly convicted, it is probable that more of the wary wicked escape than are entrapped. The crime being the outcome of the intent instead of the act, and as the intent can only be inferred from the facts in proof, it follows that there can be no certitude in criminal conviction except upon the confession of the accused. And a man candid enough to confess to what will consign him to a call cannot be considered a confirmed criminal, one depraved beyond redemption ; while one who most vehemently disclaims ever having committed a misdeed and always alert to denounce those who have, is presumably a hypocrite and liar, and a criminal in his conscience.

A father driven to desperation by the destitution of his family helps himself from the hoard of some hard-hearted old miser. Prison doors are slammed upon him *sans* ceremony He is promptly and pitilessly branded as a robber—one whose liberty would imperil the public. Another, a real wretch, robs a worthy woman of what is worth the world to her, her reputation, and snaps his fingers at the penal code. He may even be elected judge, and sentence to Sing Sing fathers who steal bread for their starving babies.

So that the records of our courts constitute no criterion of actual criminality, and until there be established on earth a tribunal of eternal justice, that may unmask the motives of men and apply to their acts the plummet of unerring judgment, let the golden rule be read between the lines of the law, and charity become a guest of the court room. Until then, set not the seal of ineffaceable infamy upon the forehead of offenders who may be such from misfortune more than malevolence, and consign them as a class of incurable moral lepers to everlasting scorn, suspicion and ostracism. Do not drive them from the doorway of society in inexpiable disgrace, and deprive them of the right to ever return. For who of humanity holds himself so holy that he can condemn a fellow creature as irrecoverably lost and hope for heaven himself without further forgiveness than he is willing to vouchsafe to others? Who would surrender his soul to the Supreme Judge upon such conditions of justice as he imposes on his erring brothers?

And who among the wisest of the world will fix the bounds between mistake and misdemeanor, indiscretion and crime, folly and felony? Who will stretch a line of human rectitude and say, those upon this side are saints and those on that are sinners, those upon this side are virtuous and those on that are vicious, those upon this side are Christians and those on that are criminals? If none, then cease this Pharisaical censoriousness that would stamp the indelible die of criminality upon every act of misconduct, and provide prison infernos for the fallen and unfortunate.

Mark well! No man is immaculate, and none, not even the meanest, can fall below the limit of human nature. And consider that there is no Holy Ghost in human nature against which an unpardonable sin can be committed, that there is more weakness than wickedness in the world, and that we never can reclaim a criminal by killing him, while perchance we can by kindness.

